# Reduction Mammoplasty Using the McKissock Technique With a Free Nipple Graft: A Safe Approach

**DOI:** 10.7759/cureus.73359

**Published:** 2024-11-09

**Authors:** José L Villarreal-Salgado, Carlos E Luna-Guerrero, Luis E Ocampo-Guzmán, Sergio E Vázquez-Lara, Harvey Y Zamora-Veliz

**Affiliations:** 1 Plastic and Reconstructive Surgery, Institute for Social Security and Services for State Workers, Zapopan, MEX; 2 General Surgery, Hospital Regional Universitario de Colima, Colima, MEX; 3 General Surgery, Institute for Social Security and Services for State Workers, Monterrey, MEX

**Keywords:** breast tissue, macromastia, mammary hypertrophy, mammoplasty, mckissock

## Abstract

Mammary hypertrophy, or macromastia, is an increase in breast tissue volume due to multiple etiologies and still unknown pathophysiology. We report the case of a 45-year-old patient with juvenile macromastia, which caused cervical pain and decreased quality of life. It was decided to perform a reduction mammoplasty with a modified McKissock technique with a free nipple graft; the resected glandular tissue was 1039 grams in the right breast and 1029 grams in the left breast. An adequate projection of the upper pole was achieved with a good aesthetic result and clinical improvement of the patient.

## Introduction

The mammary gland is mainly made up of fibroglandular tissue and fatty tissue. Mammary hypertrophy, or macromastia, is defined as a significant increase in breast tissue above normal, generating both physical and psychological symptoms [1] that lead to a decrease in the quality of life.

There are different theories of the etiology of breast hypertrophy, such as the hormonal theory, where estrogenic receptors generate a response at the level of the breast tissue, causing an increase mainly of fibrous, fatty, and, to a lesser extent, glandular tissue [1].

Breast hypertrophy is of great clinical importance due to its frequency and clinical manifestations. The volume removed can be less than 300 for mild breast hypertrophy; moderate breast hypertrophy from 300 to 700 grams; large breast hypertrophy from 700 to 1200 grams; and gigantomastia for more than 1200 grams [2].

Macromastia can generate a wide variety of symptoms, among them neck and back pain, stretch marks, and headaches, generating a great psychological impact, and each of these aspects improves with reduction surgery [3]. In the clinical case described below, it was decided to perform a reduction mammoplasty because the patient presented multiple symptoms that affected her quality of life.

## Case presentation

This is a 45-year-old female patient, with no history of diseases. The condition began at puberty with an increase in bilateral breast tissue, also reporting pain in the cervical region for 10 years as well as pain in the shoulders.

Physical examination revealed bilateral breast hypertrophy with the nipple-areola complex at 37 cm from the clavicular border, 11 cm sternal border to the nipple, and 13 cm inframammary line.

**Figure 1:**
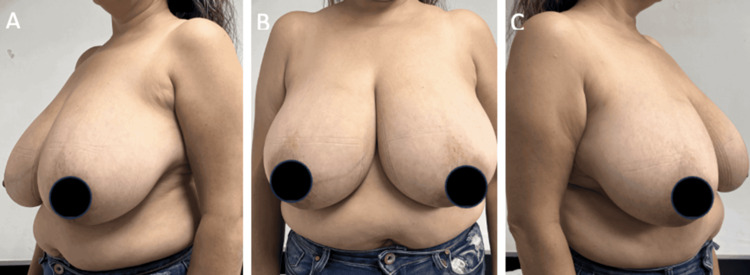
Preoperative photos A-C: lateral and frontal images

The need for surgical treatment is concluded, pre-surgical photos are taken (Figures 1A-1C), marking with wise epithelial excision pattern is performed and the patient is scheduled for reduction mammoplasty with modified McKissock technique with free nipple graft. It was decided to perform the McKissock technique to maintain adequate projection and vascularity to the NAC, performing the graft for proper nipple position and decreasing the risk of ischemia or necrosis.

**Figure 2:**
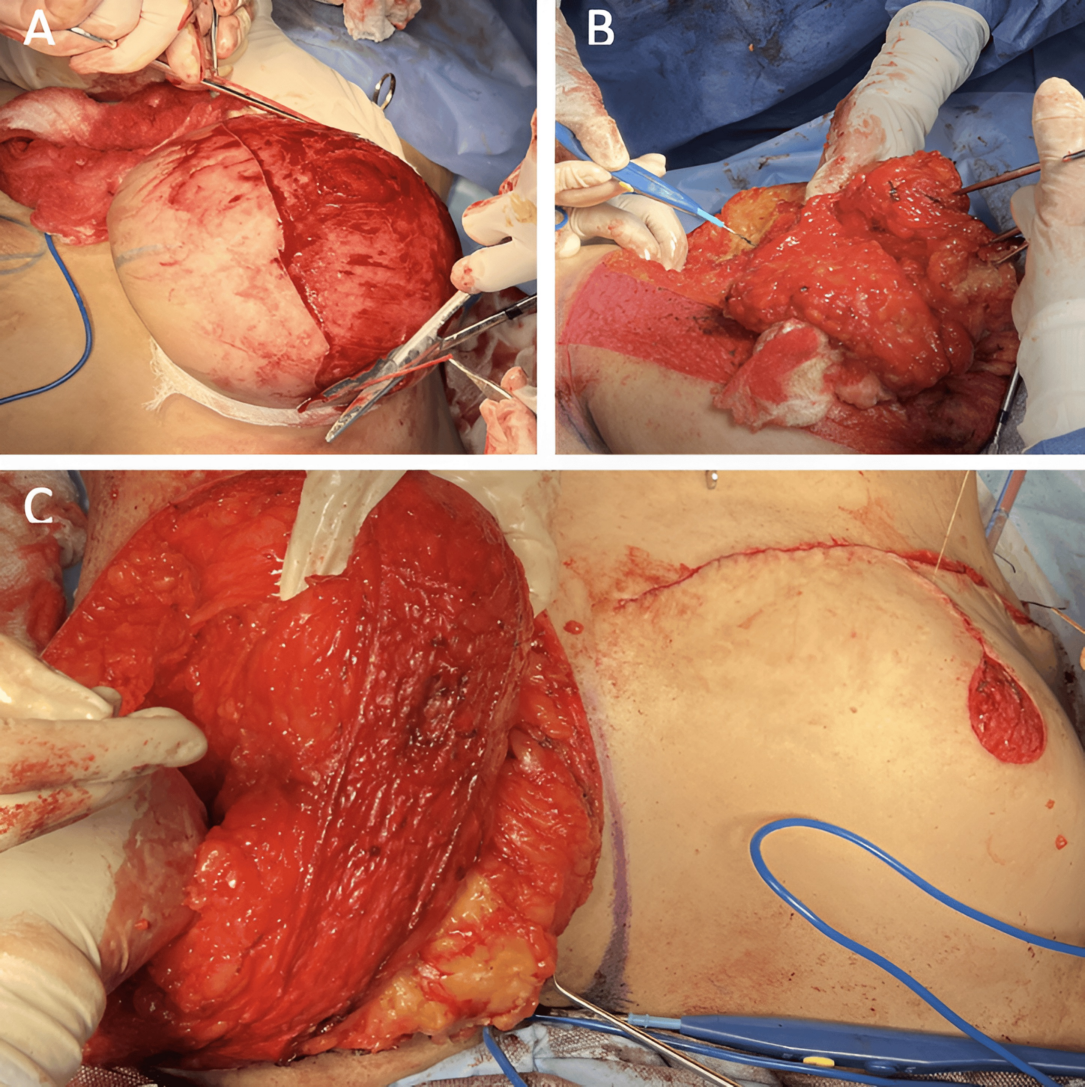
Reduction mammoplasty with McKissock technique A: nipple graft and central pedicle de-epithelization; B: resection of lateral and medial vertical pedicles; C: breast volume comparison

During surgery, nipple graft harvest is performed, followed by de-epithelization of the central pedicle and resection of the lateral and medial vertical pedicles in the right breast (Figures 2A-2C), removing 1036 g; the same procedure is performed in the left breast, removing 1040 g. The nipple-areola complex is measured at 8 cm from the sternal line and 22 cm from the clavicular border. Drainage was placed, and the graft was secured with monocryl 4-0 single stitches, dressed in gauze wrapped in the surgical field, and fixed with a tie-over technique.

**Figure 3:**
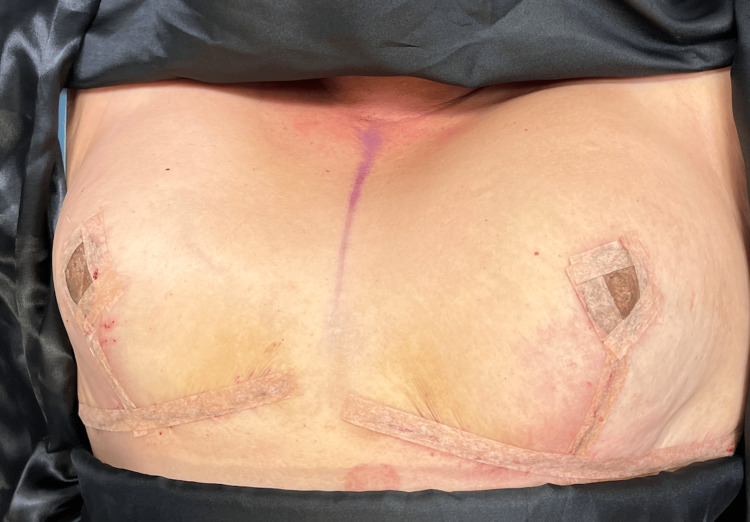
Evolution two days after surgery

In the postoperative period, with adequate evolution and without acute complications due to the intervention, discharge was decided two days after surgery (Figure 3) with analgesics and antibiotics, drains were removed, and an eighth-day appointment was scheduled where the adequate vascularization of the graft was verified and the patient no longer needed to perform wound healing, and an appointment was made for an evaluation at three months.

At the post-surgical evaluation appointment, a graft was discovered after 10 days, where adequate vascularity of the graft was observed with adequately addressed edges.

## Discussion

Breast hypertrophy is an increase in breast tissue volume. There are multiple causes, which can be secondary to physiologic conditions such as pregnancy-gestational gigantomastia (the most frequent cause) and juvenile gigantomastia [4]. Gigantomastia is defined as a distance from the sternal notch to the nipple of ≥40 cm or ≥1500 grams of tissue removed from each breast during surgery [5]. In our clinical case, the sternal notch distance was 37 cm, and the resection was just over 1000 grams.

The pathophysiology of gigantomastia depends on the type, and it is believed that it may be the result of a hormonal disorder [4] where estrogen receptors cause an increase in breast volume.

An increase in breast volume may result in postural changes that are associated with chronic back, neck, and shoulder pain [6]. Other associated symptoms include headache, rash under the breasts, and mastalgia [7]. Symptoms secondary to breast hypertrophy or gigantomastia are indications for reduction mammoplasty.

Reduction mammoplasty is a procedure in which excess breast tissue is reduced to alleviate symptoms related to macromastia, which has been shown in multiple studies to improve quality of life [8,9].

Reduction mammoplasty can be performed by multiple techniques, including lipectomy, free nipple graft, multiple pedicle designs, and skin resection patterns [10]. Reduction mammoplasty by inferior pedicle technique has been shown to confer the greatest vascular reliability; thus, it is the technique of choice for most plastic surgeons in the United States [10].

Within reduction mammoplasty techniques, bipedicled techniques have demonstrated safety in terms of flap vitality. One of the most widely used techniques is the one described by Dr. Paul Kendrick McKissock in 1977 [11], where the Strombeck technique was modified, in which, instead of performing the double horizontal pedicle, a double vertical pedicle is performed.

The fundamental objective of reduction mammoplasty is to achieve a good projection and proper sensory and vascular supply to the nipple-areola complex.

The McKissock technique was initially described for breast tissue resections up to 1000 grams; however, good results were demonstrated even in resections of more than 1000 grams [12]. The objectives of the technique focus on maintaining a vascularized central pedicle, achieving an adequate rise of the nipple-areola complex (NAC), achieving adequate projection of the breast at the upper pole, and being easily reducible and applicable [12]. In the procedure performed, the main objectives were accomplished; however, to minimize the risk of NAC perfusion, it was decided to perform a free nipple graft despite the decrease or loss of sensitivity of the nipple, making a modification in the technique.

Usually, in the McKissock technique, the nipple-areola complex remains attached to the pedicle; nevertheless, in patients with ptosed breasts, there may be vascular compromise due to excess breast length [13]. In addition, if the pedicle is inappropriately rotated, it can disrupt the vasculature of the pedicle, causing nipple necrosis [14]. In the case of our patient, it was decided to perform the modified McKissock technique with a nipple-free graft to minimize the risk of perfusion of the nipple-areola complex, since this procedure has been shown to provide a better breast shape and an adequate nipple location, decreasing the risk of vascular interruption of the pedicle [15].

## Conclusions

Breast hypertrophy most of the time is usually idiopathic in origin; however, it has been seen that hereditary factors have a very important component as well as the hormonal origin and the tendency to obesity. The mammary glands will always have a great physical, sexual, aesthetic, and psychological impact, factors that must be taken into account when carrying out a treatment, requiring most of the time multidisciplinary management to achieve a positive impact on the quality of life of patients.

Reduction mammoplasty is a surgical procedure that has implications beyond aesthetics and has a positive impact on the quality of life of patients.
